# Frequency-dependent changes in the amplitude of low-frequency fluctuations in post stroke apathy: a resting-state fMRI study

**DOI:** 10.3389/fpsyt.2025.1458602

**Published:** 2025-02-14

**Authors:** Ying Liu, Yi-Kuang Hsien, Wenlong Su, Zhiqing Tang, Hui Li, Junzi Long, Xingxing Liao, Hao Zhang

**Affiliations:** ^1^ School of Rehabilitation, Capital Medical University, Beijing, China; ^2^ Beijing Bo’ai Hospital, China Rehabilitation Research Center, Beijing, China; ^3^ Cheeloo College of Medicine, Shandong University, Jinan, Shandong, China

**Keywords:** post stroke apathy, resting state fMRI, amplitude of low-frequency fluctuation, fractional amplitude of low-frequency fluctuation, superior temporal gyrus, middle frontal gyrus

## Abstract

**Background:**

Apathy is a prevalent psychiatric condition after stroke, affecting approximately 30% of stroke survivors. It is associated with slower recovery and an increased risk of depression. Understanding the pathophysiological mechanisms of post stroke apathy (PSA) is crucial for developing targeted rehabilitation strategies.

**Methods:**

In this study, we recruited a total of 18 PSA patients, 18 post-stroke non-apathy (NPSA) patients, and 18 healthy controls (HCs). Apathy was measured using the Apathy Evaluation Scale (AES). Resting-state functional magnetic resonance imaging (rs-fMRI) was utilized to investigate spontaneous brain activity. We estimated the amplitude of low-frequency fluctuation (ALFF) across three different frequency bands (typical band: 0.01–0.08 Hz; slow-4: 0.027–0.073 Hz; slow-5: 0.01–0.027 Hz) and the fractional amplitude of low-frequency fluctuation (fALFF).

**Results:**

Band-specific ALFF differences among the three groups were analyzed. Significant differences were found in the typical band within the left lingual gyrus, right fusiform gyrus, right superior temporal gyrus (STG), and left insula. In the slow-4 band, significant differences were observed in the left middle frontal gyrus (MFG) and right STG. In the slow-5 band, significant differences were identified in the left calcarine cortex and right insula. For fALFF values, significant differences were found in the left lingual gyrus and right thalamus. Moreover, positive correlations were observed between AES scores and the ALFF values in the right STG (r = 0.490, *p* = 0.002) in the typical band, left MFG (r = 0.478, *p* = 0.003) and right STG (r = 0.451, *p* = 0.006) in the slow-4 band, and fALFF values of the right thalamus (r = 0.614, *p* < 0.001).

**Conclusion:**

This study is the first to investigate the neural correlates of PSA using voxel-level analysis and different ALFF banding methods. Our findings indicate that PSA involves cortical and subcortical areas, including the left MFG, right STG, and right thalamus. These results may help elucidate the neural mechanisms underlying PSA and could serve as potential neuroimaging indicators for early diagnosis and intervention.

## 1 Introduction

Apathy is characterized by a measurable decline in goal-directed behaviors across the cognitive, emotional, and social dimensions of an individual’s life (1). It is a common neuropsychiatric symptom after stroke, which affects approximately 30% of stroke survivors (2, 3). Post-stroke apathy (PSA) is associated with functional disabilities, slower recovery, cognitive deficits and an increased risk of subsequent depression (4–6). However, the pathophysiological mechanisms that contribute to apathy in stroke patients are yet to be fully elucidated. Understanding these mechanisms could facilitate the development of more effective, targeted rehabilitation strategies for PSA.

Resting-state functional magnetic resonance(rs-fMRI), which assesses spontaneous fluctuations in blood-oxygen-level-dependent signals, serves as a robust non-invasive method for examining brain functional connectivity (7, 8). During rest, the brain is still active, resulting in slow fluctuations of blood flow that is seen as a marker of spontaneous brain activation (9). The amplitude and frequency of these fluctuations correlate with emotional regulation (10). The amplitude of low-frequency fluctuations (ALFF) quantifies the spontaneous activity amplitude of brain regions during resting state by calculating the average square root of the power spectral density within a specific low-frequency range (0.01-0.08Hz) (11). Further, the typical frequency bands can be divided into the following ranges: slow-5(0.01–0.027Hz), slow-4(0.027–0.073Hz), slow-3(0.073–0.198Hz), and slow-2 (0.198–0.25Hz) (9, 12). Studies indicate that various neural oscillation frequencies within the brain may exhibit sensitivity to activity across different brain regions. The slow-5 and slow-4 bands are primarily related to gray matter, whereas slow-3 and slow-2 bands are associated with white matter and can be affected by aliasing with respiratory and cardiac signals (9). The fractional amplitude of low-frequency fluctuations(fALFF) is a method that calculates the ratio of the power spectrum within the low-frequency range to the power spectrum across the entire frequency range (13). It also measures spontaneous neural activity, and is often preferred because it standardizes the power spectra and is robust against physiological noise (13). These rs-fMRI indices are widely utilized in the study of neurological disorders, such as Parkinson’s disease, Alzheimer’s disease, and stroke (14–16).

Neuroimaging studies have consistently linked the apathetic syndrome to disruptions in specific medial frontal cortex and subcortical structures, particularly the anterior cingulate cortex, medial orbitofrontal cortex, and ventral striatum (17, 18). These areas are integral parts of the brain’s reward system. Emerging physiological evidence indicates that reward processing is altered in apathetic patients, making them less sensitive to rewarding outcomes (19, 20). Previous studies have primarily associated apathy with abnormal activity in various brain regions, including the frontal lobe and the cingulate gyrus. For example, Parkinson’s patients with apathy show reduced ALFF in the right anterior cingulate gyrus in the slow-5 band and decreased fALFF in the right middle frontal gyrus in the typical band (21). Another study indicated decreased ALFF in left orbital middle frontal gyrus and bilateral superior frontal gyrus (22). Only one study has investigated the fALFF, finding significant differences in the left middle temporal regions, right anterior and middle cingulate regions, middle frontal region, and cuneus region in PSA patients (23). However, to date, no studies have explored changes in brain regions across different frequency bands in PSA patients. It is critical for elucidating the pathophysiological mechanisms underlying apathy after stroke.

In this study, we utilize ALFF and fALFF to investigate the changes in spontaneous brain activity during resting state in PSA patients. Based on prior study, we hypothesize that PSA patients will exhibit alterations in ALFF within the frontal lobes and subcortical region. Identifying abnormalities in spontaneous brain activity in PSA patients could elucidate potential therapeutic targets, thereby improving our understanding and management of PSA.

## 2 Methods

### 2.1 Participants

The inclusion criteria for this study were as follows: (1) a confirmed diagnosis of stroke with a disease duration of more than one month; (2) a diagnosis of apathy based on established consensus criteria, with an Apathy Evaluation Scale (AES) score greater than 38 (24, 25); (3) ages between 18 and 75 years; (4) were right-handed; (5) willing to participate to the study, and signed a written informed consent. The exclusion criteria were: (1) presence of severe liver or kidney diseases or a history of tumors; (2) contraindication for MRI; (3) refusal to participate in the study. This study adheres to the Helsinki Declaration and has been approved by the Medical Ethics Committee of the China Rehabilitation Research Center.

### 2.2 Neuropsychological evaluation

Apathy was assessed by the Apathy Evaluation Scale (AES), which includes 18 items evaluating the behavioral, cognitive, and emotional dimensions of apathy (26). The AES has a cutoff score of >38, with scores ranging from 18 to 72; higher scores indicate greater apathy. Depression was assessed by Hamilton Depression Rating Scale (HAMD). HAMD is the most commonly used depression scale because it has a high specificity to assess the severity of depression symptoms. The Cronbach’s α score of HAMD-24 is 0.88, and the κ-score is 0.92 (27). Cognitive function was assessed using the Mini-Mental State Examination (MMSE), a widely utilized tool for evaluating cognitive function globally (28, 29). The total cognition score ranges from 0 to 30 (30).

Neurological functional status was assessed using the National Institutes of Health Stroke Scale (NIHSS) and Barthel index. The NIHSS is a standardized tool used to quantify stroke severity (31). The total NIHSS score ranges from 0 to 42, with higher totals signifying greater stroke impact. The Barthel index was utilized to evaluate activities of daily living. The Barthel index includes ten self-care items including grooming, bathing, feeding, toilet use, dressing, walking, transferring from bed to chair, stair climbing, bowel control, and bladder control (32).

### 2.3 Imaging data acquisition

Participants underwent both T1 and rs-fMRI scanning. The data were collected on a Philips Ingenia 3T MRI scanner equipped with a 32-channel head coil. High resolution T1-weighted anatomical images were acquired (TR = 7.13ms, TE =3.22ms; FOV = 256 × 256 mm^2^; flip angle = 7°; 256 × 256 matrix; 192 slices; voxel size = 1 × 1 × 1 mm^3^). Rs-fMRI were acquired using a gradient echo planar imaging (EPI) sequence (TR = 2000ms, TE =30ms; FOV = 224 × 224 mm2; flip angle = 90°; 64 × 64 matrix; 32 slices; voxel size = 3.5 × 3.5 × 4.35 mm^3^). Participants were required to keep still and awoke during the entire scanning.

### 2.4 Data preprocessing and analysis

Data preprocessing was performed using the DPARSF (http://rfmri.org/DPARSF) toolkit based on the MATLAB 2021b (33). Preprocessing includes the following steps: (1) Conversion of DICOM format to NFITI format; (2) Removal of the first ten time points; (3) Time layer and head motion correction; (4) Each participant’s fMRI images were co-registered with their T1 images. Subsequently, the DARTEL method was employed to segment the T1 images into gray matter, white matter, and cerebrospinal fluid, followed by spatial transformation of the segmented gray matter probability maps. The same transformation parameters were then applied to the corresponding fMRI images; (5) Spatial smoothing was performed using a 8-mm full width at half maximum (FWHM) Gaussian kernel to reduce noise and enhance the reliability of the signal; (6) Detrend; (7) regression of head motion, brain white matter signal, cerebrospinal fluid signal, and global signal as covariates. In this study, the rs-fMRI data for subjects with head motion displacement >2.5mm or rotation >2.5° in any axis were discarded. Four participants were consequently excluded from the study due to head movement. A total of 18 PSA patients, 18 post stroke non-apathy (NPSA) patients and 18 health controls (HC) were finally included in the following analysis.

The typical bands (0.01-0.08Hz) (11), slow-5(0.01–0.027Hz) and slow-4(0.027–0.073Hz) were extracted. and the fALFF value was obtained by dividing the sum of ALFF values in this frequency band by the sum of amplitudes in the full frequency band. Normalization was performed by dividing each voxel’s ALFF or fALFF value within the specified frequency band by the brain-wide mean, yielding normalized metrics (mALFF and mfALFF) to quantify relative low-frequency fluctuations for statistical analysis.

### 2.5 Statistical analysis

The age, gender and years of education were compared using analysis of variance (ANOVA) in the PSA, NPSA, and HC group. The chi-square test was used to analyze the disease types and lesion sides. The duration of disease, as well as the AES, HAMD, MMSE, NIHSS, and Barthel Index scores, between the PSA and NPSA groups were analyzed using either the Mann-Whitney test or the t-test, depending on the distribution and nature of the data. The above data were analyzed by SPSS (version 25.0, Armonk, NY, USA). Statistical significance was set at *p*<0.05. Sex, age, and head motion were used as covariates in the ANOVA analysis to compare ALFF and fALFF values among the three groups. *Post-hoc* pairwise t-tests with Bonferroni correction were conducted to evaluate group differences in ALFF and fALFF values among the PSA, NPSA, and HC groups. Spearman correlation analysis was performed to assess the relationship between ALFF, fALFF values, and AES scores within the regions that showed significant differences between the PSA and NPSA groups.

## 3 Results

### 3.1 Demographic and clinical characteristics

In this study, 54 participants were selected for the present study, including 18 PSA patients, 18 NPSA patients and 18 HCs. A summary of the participants’ demographic and clinical data is presented in **Table 1**. There were no significant differences in gender (*p* =0.758), age (*p* = 0.779), education (*p* = 0.271), diagnosis (*p* = 0.735), lesion hemisphere (*p* =0.423) among three group. PSA group and NPSA group showed no significant difference in MMSE (*p* =0.158), HAMD (*p* =0.346), NHISS (*p* =0.447), BI (*p* =0.686). Compared with the NPSA group, the PSA patients exhibited significantly higher AES scores (*p* < 0.001).

**Table 1:** Participants’ demographic and clinical data.

	PSA	NPSA	HC	F/T
Gender				
male	14	12	13	0.758
female	4	6	5	
Age	52.33 ± 12.319	54.50 ± 10.350	54.28 ± 6.890	0.779
Education (year)	14.16 ± 2.007	14.26 ± 1.643	14.41 ± 1.561	0.271
Diagnosis				
cerebral infarction	10	11	/	0.735
cerebral hemorrhage	8	7	/	
Hemisphere				
left	15	13	/	0.423
right	3	5	/	
Disease duration (month)	1.67 ± 0.514	1.56 ± 0.591	/	0.552
AES	49.56 ± 5.426	20.28 ± 4.443	/	<0.001
MMSE	22.72 ± 4.599	24.94 ± 4.646	/	0.158
HAMD	22.83 ± 1.855	23.67 ± 3.199	/	0.346
NHISS	4.72 ± 2.396	5.50 ± 3.552	/	0.447
BI	60.00 ± 22.752	56.94 ± 22.172	/	0.686

AES, Apathy Evaluation Scale; MMSE, Mini-Mental State Examination; HAMD, Hamilton Depression Rating Scale; NHISS, National Institutes of Health Stroke Scale; BI, Barthel index.

### 3.2 ALFF and fALFF values in the PSA, NPSA, and HC groups

**Figure 1:**
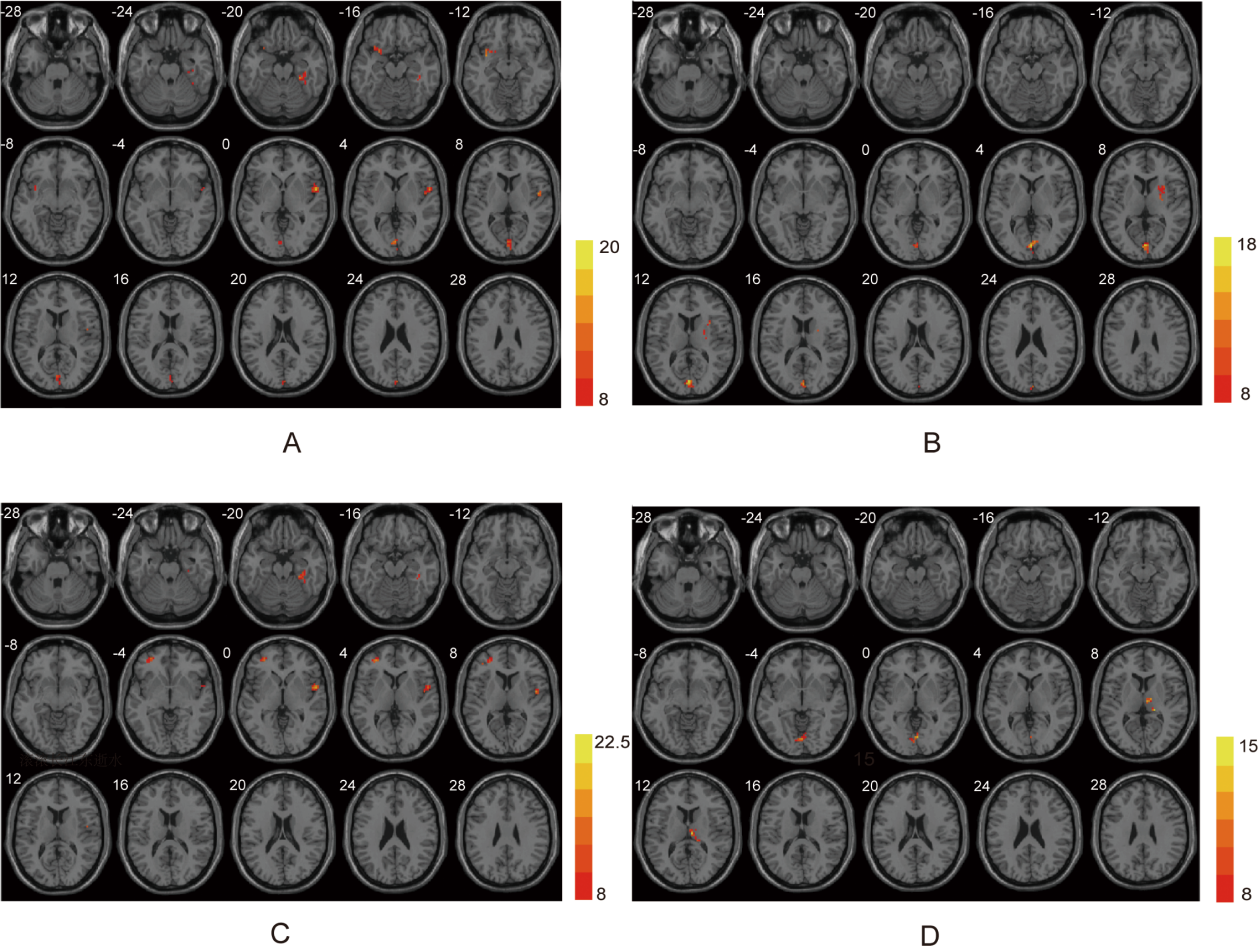
ANOVA differences in different frequency bands. **(A)** Brain regions showing typical band differences among PSA, NPSA and HC groups. **(B)** Brain regions showing slow-5 band differences among three groups. **(C)** Brain regions showing slow-4 band differences among three groups. **(D)** Brain regions showing fALFF differences among three groups.

**Figure 2:**
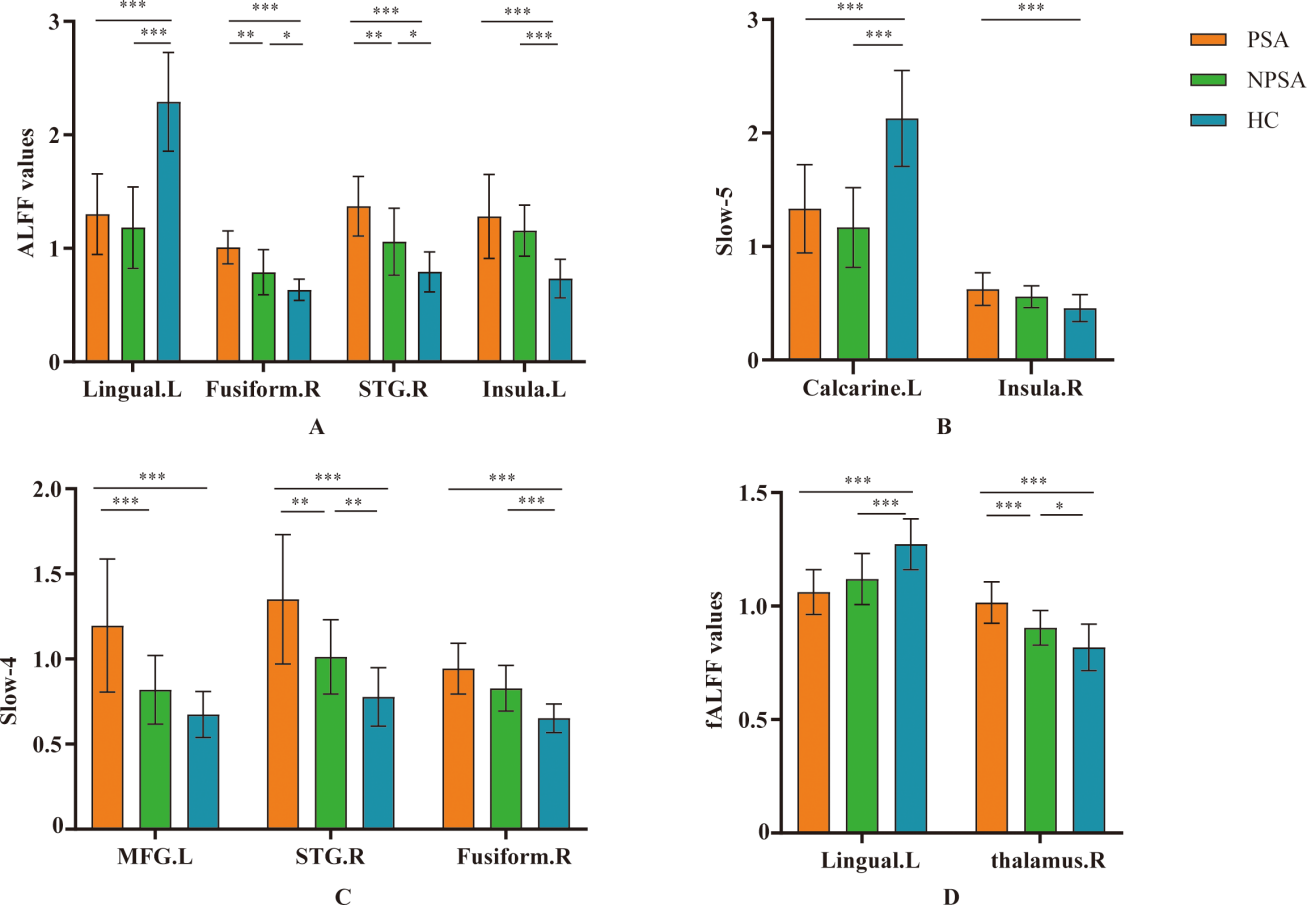
*Post-hoc* comparisons of ALFF between each pair of three groups. Between-group differences of ALFF in **(A)** typical band, **(B)** slow-5 band, **(C)** slow-4 band, and **(D)** fALFF. *p < 0.05, **p < 0.01, ***p < 0.001. L, left; R, right. MFG, middle frontal gyrus; STG, superior temporal gyrus.

In typical band (0.01–0.08 Hz), there are significant differences in left lingual, right fusiform, right superior temporal gyrus (STG), left insula among three group (voxel *p*<0.001, cluster *p*<0.05, GRF corrected, cluster size >30 voxels) (**Table 2**, **Figure 1A**). *Post-hoc* analyses showed increased ALFF values in the right fusiform and right STG in PSA group when compared with both NPSA and HC groups (*p*<0.05) (**Figure 2A**).

**Table 2:** Covariance results of ALFF values among the PSA, NPSA and HC groups.

Brain regions	Peak value			Voxels	Size (mm^^3^)	F values
	X	Y	Z			
Typical band						
Lingual.L	0	-81	6	50	1350	15.03
Fusiform.R	36	-33	-18	38	1026	21.28
STG.R	54	3	0	37	999	19.73
Insula.L	-30	15	-18	30	810	15.09
Slow-5						
Calcarine.L	0	-81	12	69	1863	19.68
Insula.R	33	12	12	30	810	12.42
Slow-4						
MFG.L	-30	48	3	41	1107	16.55
STG.R	54	3	0	35	945	19.66
Fusiform.R	36	-36	-18	34	918	20.14
fALFF						
Lingual.L	0	-78	-3	28	756	14.14
Thalamus.R	6	-18	12	23	621	15.75

In slow-5 band (0.01–0.027 Hz), there are significant differences in left calcarine and right insula among three group (voxel *p*<0.001, cluster *p*<0.05, GRF corrected, cluster size >30 voxels) (**Table 2**, **Figure 1B**). However, there are no significant difference in PSA group when compared with both NPSA and HC groups by *post-hoc* analyses (*p*<0.05) (**Figure 2B**).

In slow-4 band (0.027–0.073Hz), there are significant differences in left middle frontal gyrus (MFG), right STG, right fusiform among three group (voxel *p*<0.001, cluster *p*<0.05, GRF corrected, cluster size >30 voxels) (**Table 2**, **Figure 1C**). *Post-hoc* analyses exhibited increased left MFG and right STG in PSA group when compared with both NPSA and HC groups (*p*<0.05) (**Figure 2C**).

For fALFF values, there are significant differences in left lingual and right thalamus among three group (voxel *p*<0.001, cluster *p*<0.05, GRF corrected, cluster size >30 voxels) (**Table 2**, **Figure 1D**). *Post-hoc* analyses exhibited increased right thalamus in PSA group when compared with both NPSA and HC groups (*p*<0.05) (**Figure 2D**).

### 3.3 ALFF and fALFF values associated with clinical scores in stroke patients

**Figure 3:**
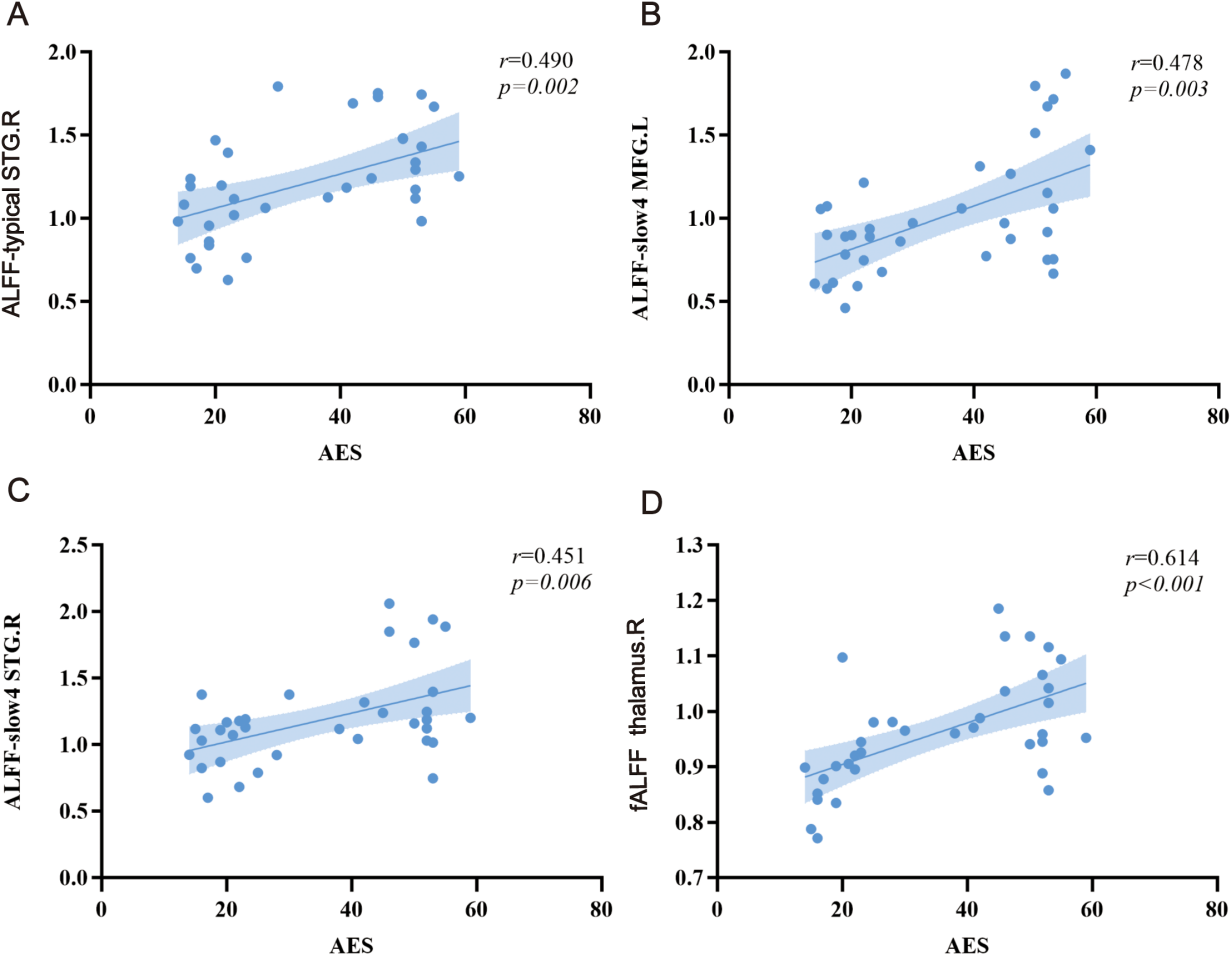
Relationship between ALFF alterations and apathy scores. **(A)** typical band, **(B, C)** slow-4, and **(D)** fALFF. L, left; R, right. MFG, middle frontal gyrus; STG, superior temporal gyrus.

The ALFF values of right STG (r = 0.490, *p* = 0.002) in typical band are positively correlated with the AES scores in stroke patients. The ALFF values of left MFG (r = 0.478, *p* = 0.003) and right STG (r = 0.451, *p* = 0.006) in slow-4 band are positively correlated with the AES scores in stroke patients. The fALFF values of right thalamus is positively correlated with the AES scores in stroke patients (r = 0.614, *p* < 0.001) (**Figure 3**).

## 4 Discussion

This study utilizes different frequency bands of ALFF to investigate local functional abnormalities in PSA patients. Our findings reveal that the primary brain regions exhibiting abnormalities in PSA patients are concentrated in the frontal and temporal lobes, as well as the thalamus. Moreover, the ALFF values in the left MFG, right STG, and right thalamus are positively correlated with apathy scores.

The MFG, a core component of the dorsolateral prefrontal cortex (DLPFC), plays a critical role in regulating goal-directed behavior, motivation, and emotional responses, all of which are often impaired in apathy (34, 35). Dysfunction in the MFG has been strongly linked to apathy, a condition characterized by a quantitative reduction in voluntary, goal-directed behaviors. This is particularly evident in patients with focal prefrontal cortex lesions, where decreased reactivity to emotional and reward-related stimuli leads to deficits in decision-making. These deficits hinder the ability to accurately evaluate the consequences of actions within emotional and affective contexts, resulting in diminished motivation and impaired goal-directed behavior (36–38). Apathy is further characterized by a significant decrease in the motivation to set and pursue goals, independent of cognitive impairment, emotional distress, or depression (39). Structural imaging studies have consistently shown reduced gray matter volumes in individuals with apathy, particularly in the frontal and temporal lobes (40). In conditions such as cognitive impairment and Alzheimer’s disease, apathy has also been associated with increased white matter lesions and atrophy in frontal gray matter (41–43). Hypoactivity in frontal lobe regions, including the DLPFC, has been identified as a major contributor to apathy following stroke (44). In this study, we observed increased ALFF values in the left MFG, consistent with its critical role in integrating cognitive and motivational processes. This finding aligns with prior research linking DLPFC activity and connectivity with apathy severity. Reduced activity and connectivity within the DLPFC, including the MFG, has been associated with higher levels of apathy, particularly in tasks involving working memory, attentional processing, and motivational regulation. These findings underscore the centrality of the MFG in the neural mechanisms of apathy, highlighting its role in bridging cognitive and emotional processes to sustain goal-directed behavior (45).

The temporal lobe plays a critical role in emotion, memory, and social behavior, with STG serving as a key component of this region (46–48). Structural abnormalities in the temporal lobe, such as reduced inferior-temporal cortical thickness, have been identified as significant risk factors for apathy, particularly in Alzheimer’s disease and mild cognitive impairment (49, 50). Functional studies have further highlighted the STG’s essential role in the neural mechanisms of apathy. For instance, reduced functional connectivity between the right STG and regions such as the right supramarginal gyrus and the left precuneus has been significantly associated with self-reported apathy symptoms in older adults (51). These disrupted connections likely impair the integration of social and emotional information, contributing to decreased motivation and the manifestation of apathy. In stroke patients, functional abnormalities in the STG are further supported by changes in regional cerebral blood flow (rCBF). Okada et al. demonstrated that apathetic stroke patients exhibited significantly reduced rCBF in the right DLPFC and left frontotemporal regions, with apathy severity negatively correlated with rCBF in these areas (44, 51). This indicates that the STG not only plays a localized functional role but also influences motivational states through its broader network connections. Our findings further confirm the importance of the STG in PSA. PSA patients showed significantly increased ALFF values in the right STG across both the typical band and the slow-4 band. This increase in ALFF may reflect compensatory changes in local neural activity to counterbalance network dysfunction caused by disrupted functional connectivity. This result aligns with studies of first-episode, drug-naive major depressive disorder patients, who also exhibited significant increases in ALFF values in the right STG, suggesting that the STG may play a similar role in regulating emotion and motivation across different pathological conditions (52). Additionally, growing evidence suggests that post-stroke apathy arises not from damage to a single brain region but from the disconnection of a complex functional network (53). As a hub for integrating auditory, emotional, and social signals, the STG is critical for maintaining the functional coherence of these networks. Disruptions in STG activity and its connectivity patterns are likely to propagate through the network, leading to widespread impairments in emotional and motivational regulation.

In addition, beyond the changes in ALFF values in cortical regions, spontaneous activity in subcortical regions also changed in PSA patients. Our results showed that right fusiform and right thalamus exhibited increased ALFF values in PSA patient. A study indicated that patients with apathetic depression had lower functional connectivity between the nucleus accumbens and the thalamus (54). Apathy may be a prominent feature of thalamic strokes, particularly those involving the territory of the paramedian arteries, including the dorsomedian and intralaminar thalamic nuclei (55). The thalamus plays a crucial role in regulating emotional and behavioral processes due to its extensive connections, which are critical for emotional and motivational regulation. Research has shown that thalamic damage can trigger symptoms of apathy (55, 56). Our study further confirms the important role of the thalamus in apathy.

Finally, several brain regions, including the right STG, the left MFG, and the right thalamus, were found to be positively correlated with apathy score. These results suggest that PSA is closely related to functional changes in several brain regions, involving multiple aspects of emotion, motivation, and cognition (57). Alterations in right STG, left MFG, and right thalamic function may work together to cause a decrease in patients’ levels of emotion and motivation. In the future, ALFF changes in PSA patients at different stages of recovery could be tracked to understand the relationship between functional changes in brain regions and the evolution of apathy symptoms. Additionally, targeting these apathy-related brain regions with transcranial magnetic stimulation or other neuromodulation techniques could potentially improve symptoms. Combining various imaging techniques, such as electroencephalography, could further elucidate the specific mechanisms through which these brain regions regulate emotion and motivation.

There are a couple of limitations to our findings. First, the relatively small sample size of 54 participants may limit the reliability and statistical power of our findings. This limitation underscores the need for future studies with larger cohorts to validate and extend these results. Second, although our study focused on abnormal functional changes in specific brain regions using ALFF and fALFF at the voxel level, it did not explore the interactions within and between functional brain networks. Future studies could incorporate additional analytical approaches, such as functional connectivity analysis, to provide a more comprehensive understanding of the overall abnormalities in midbrain networks among PSA patients. This may offer deeper insights into the underlying pathological mechanisms of PSA. Third, we did not consider the different symptom dimensions of apathy. Further analysis is necessary to assess the relationship between different dimensions of apathy and brain function.

## 5 Conclusion

In conclusion, this is the first study to investigate the neural correlates of PSA based on voxel level using different ALFF banding methods. These findings reveal that PSA patients mainly involve cortical and subcortical areas including left MFG, right STG, and right thalamus. This may help to elucidate the neural mechanisms of PSA and may be a potential neuroimaging indicator for early diagnosis and intervention in PSA.

## Data Availability

The raw data supporting the conclusions of this article will be made available by the authors, without undue reservation.
